# Viral glycoprotein-mediated entry and antibody-mediated immunity in HIV-1 and SARS-CoV-2 infection

**DOI:** 10.3389/fimmu.2025.1733684

**Published:** 2026-01-09

**Authors:** Michael W. Grunst, Wenwei Li, Walther Mothes

**Affiliations:** Department of Microbial Pathogenesis, Yale University School of Medicine, New Haven, CT, United States

**Keywords:** Fc-mediated immune effector functions, HIV-1, neutralizing antibodies, SARS-CoV-2, virus entry

## Abstract

Enveloped viruses such as Human Immunodeficiency Virus (HIV-1) and Severe Acute Respiratory Syndrome Coronavirus 2 (SARS-CoV-2) have caused some of the deadliest pandemics in human history. These viruses utilize Class 1 viral fusion glycoproteins to bind their host receptor and subsequently fuse the virus and host cell membranes to mediate entry. Viral fusion glycoproteins are prominent antigens on the surface of virions and are essential for the virus life cycle. Therefore, they are a primary target for the humoral immune system and the basis for the design of vaccines. Antibodies which target viral fusion glycoproteins can neutralize viral infectivity and activate the immune system in several distinct ways. In this review, we compare mechanisms of how class 1 viral fusion glycoproteins mediate viral entry and cover diverse ways in which antibodies targeting these glycoproteins can neutralize viruses and activate the immune system to clear virus-infected cells.

## Introduction

Viruses with high pandemic potential, including the human immunodeficiency virus (HIV-1) and severe acute respiratory syndrome coronavirus 2 (SARS-CoV-2) utilize class 1 membrane fusion proteins [envelope glycoprotein (Env), and Spike respectively] to mediate entry into host cells. Being prominent antigens on the surface of virions and infected cells, class 1 membrane fusion proteins are a critical component of vaccine development. Currently, no vaccine exists which reliably protects against HIV-1 acquisition, and no widely implementable cure strategy exists. While effective vaccines exist for SARS-CoV-2, they need frequent updating as strains with new mutations continuously emerge with increased resistance (1–3). Further, broader protection may be beneficial in the case of zoonotic transmission. Alarmingly, three deadly betacoronavirus zoonotic outbreaks occurred within a 20-year span (reviewed by (4)). A deep understanding of how these class 1 virus fusion proteins mediate entry into cells, and how antibodies target them, is therefore critical for public health efforts.

HIV-1 and SARS-CoV-2 class 1 viral fusion proteins utilize similar mechanisms to fuse virus and host-cell membranes (reviewed by (5–9)). Class 1 viral fusion proteins first engage host cell receptors, then undergo a large conformational change to 1) insert N-terminal or N-proximal fusion peptides into the host membrane and 2) refold to pull the virus and host membranes together. The extension and refolding has been described as a “cast-and-fold” mechanism (8). The final conformation of the class 1 fusion protein after refolding is called the “postfusion” conformation that features a characteristic 6-helix bundle. It is thought that formation of the highly stable 6-helix bundle provides the energy to overcome the electrostatic repulsions between the virus and host cell lipid membranes and to create disruptions in the lipid bilayer. These disruptions in the bilayer while in proximity can result in mixing of the outer and inner membrane leaflets and eventually the formation of a fusion pore, through which the viral contents enter the cell.

This review will focus on membrane fusion mediated by two distinct viral fusion proteins, HIV-1 Env and SARS-CoV-2 Spike, along with antibody-mediated neutralization mechanisms which block their function. Recent methodologies have arisen which have provided unique windows into viral entry, as well as novel ways antibodies inhibit the membrane fusion process. Altogether, we hope that through a deep understanding of these processes progress can be made to design more effective immunogens to elicit broad protection.

## Membrane fusion mediated by class 1 viral fusion proteins

### Common themes: virus attachment and membrane fusion

**Figure 1:**
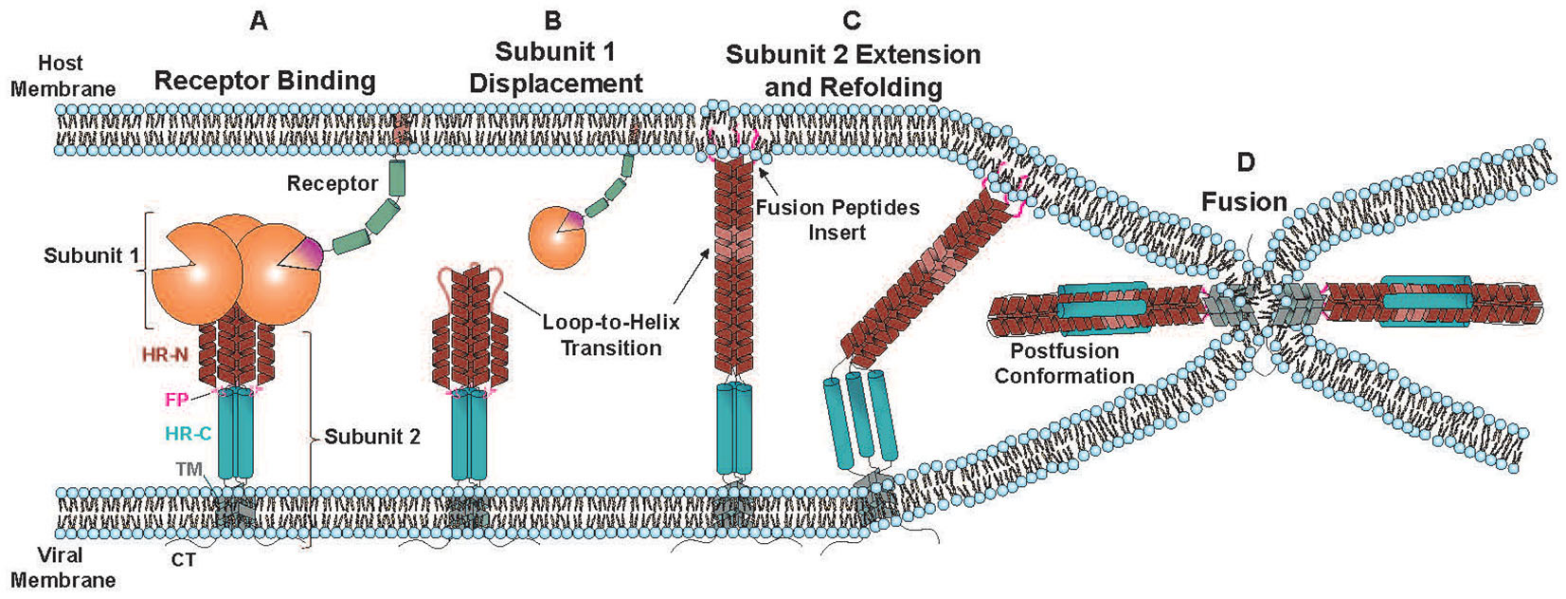
General Schematic of membrane fusion mediated by Class 1 viral fusion proteins, such as HIV-1 Env and SARS-CoV-2 Spike. FP: fusion peptide, HR-N: N-terminal heptad repeat, HR-C: C-terminal heptad repeat, TM: transmembrane domain, CT: Cytoplasmic Tail. Different class 1 viral fusion proteins will vary in size and details of these mechanisms.

Class 1 viral fusion proteins are comprised of two major subunits: gp120/gp41 for HIV-1 Env, S1/S2 for SARS-CoV-2 Spike. During biosynthesis, the viral fusion proteins are cleaved by host proteases such as furin into two subunits which are held together in a meta-stable state. Subunit 1 (HIV-1 Env gp120, SARS-CoV-2 Spike S1) mediates engagement of the host receptors and “caps” the metastable subunit 2. Subunit 1 attachment to host receptors is (often) the first event in viral entry (**Figure 1A**). The host receptors utilized for entry play a major role in viral tropism, dissemination, pathogenesis, and transmission. Discovering which host proteins act as receptors and determining their detailed molecular interactions is highly prioritized when a new virus emerges (10, 11). This step is a critical window for the host antibodies to interfere with the virus life cycle and is therefore the basis of many vaccine strategies.

After attachment mediated by subunit 1, subunit 2 of class 1 viral fusion proteins (HIV-1 Env gp41, SARS-CoV-2 Spike S2) drives the membrane fusion process. Subunit 2 contains stored energy from biosynthesis and is inherently metastable after a proteolytic “priming” step (12). Upon triggering from environmental cues (i.e. low pH, release of subunit 1), subunit 2 undergoes a large conformational change to insert fusion peptides (FP) into the host membrane and refold to pull the virus and host membranes into proximity. The energy released by subunit 2 refolding may help overcome the charge repulsion of opposing phospholipid membranes and the energy activation barriers of membrane fusion intermediates (13, 14). Although seemingly intuitive, exactly how the energy released from Subunit 2 refolding translates into membrane fusion intermediates is not well known.

Common themes in class-1 mediated membrane fusion are the general architecture of subunit 2, an N-terminal or N-proximal fusion peptide, and the rearrangement of subunit 2 from the prefusion to postfusion state that contains a characteristic 6-helix bundle (**Figure 1**). Subunit 2 is comprised of a fusion peptide, heptad-repeat N-terminal domain (HR-N), heptad-repeat C-terminal domain (HR-C), a transmembrane domain (TM), and a cytoplasmic tail (C-tail). The N-terminal fusion peptides of subunit 2 are comprised of highly conserved hydrophobic residues. The prefusion HR-N domain contains alpha-helical domains separated by flexible loop regions. Upon attachment to the host receptor and displacement of subunit 1 (**Figures 1A, B**), the flexible loop regions of subunit 2 undergo a “spring-loaded loop-to-helix” transition, thereby causing HR-N and the N-terminal fusion peptides to extend towards the target cell membrane (**Figure 1C**) (12, 15, 16). The fusion peptides at the N-terminus of the extended HR-N domains insert into the target cell membrane, thereby linking the virus and target cell membranes by subunit 2. This extended conformation of subunit 2 is known as the “prehairpin intermediate”. These prehairpin intermediates are unstable; the HR-C domains collapse back on the central HR-N domains to form a highly stable 6-helix bundle known as the postfusion conformation (the “hairpin”, **Figure 1D**). The fusion peptides in the target cell membrane and the TM in the virus membrane also form associations in the postfusion conformation (17). Upon collapse of the prehairpin intermediate, the virus and target cell membranes are distorted and pulled into proximity (18). A series of membrane fusion intermediates occur during this process of subunit 2 refolding before formation of a fusion pore.

Class 1-mediated membrane fusion may occur through different intermediate pathways (19–23), and is canonically thought to begin with protrusions of the lipid bilayer into “nipple-like” conformations or protrusions (21, 23). The tips of these protrusions may contain patches of partially exposed hydrophobic lipid tails on the outer leaflets. These hydrophobic lipid tails on the opposing membranes can begin mixing to form a “hemifusion stalk” (21). The stalk may then expand and allow mixing of the inner leaflet hydrophobic tails to make an extended hemifusion diaphragm, which finally collapses into a fusion pore (6, 13, 23). It is also possible that hemifusion stalk could transition into a pore without the formation of the extended hemifusion diaphragm (21, 23). Another fusion pathway called the “rupture-insertion” pathway occurs when a ruptured membrane spontaneously inserts into an in-tact membrane to form a hemifusion structure (19). CryoET analysis of fusion mediated by influenza HA (another class 1 viral fusion protein) with liposomes demonstrated that the preferred pathway to form hemifusion structures depends on the lipid composition (i.e. cholesterol content) (19). From hemifusion intermediates, the process progresses to a flickering fusion pore, which can still be reversible, to a widening fusion pore (24). Once a larger fusion pore is formed, the virus can release its genomic contents into the host cell and begin the replication process. Intermediate steps in this dynamic process have been observed in cryoET structural studies and modelled by molecular dynamics simulations (18, 19, 25–29). The molecular details of the prefusion-to-postfusion transition are also inferred through high resolution structures of the static prefusion and postfusion conformations using single particle cryoEM or Xray crystallography. Further developments in cryoEM techniques and molecular dynamics are needed to capture these processes in more detail.

Class 1 viral fusion glycoproteins are the most prominent antigen on the surface of enveloped viruses and therefore evolved a variety of mechanisms to evade the immune system. First, class 1 viral fusion proteins contain regions of high sequence variability, particularly in subunit 1. For HIV-1 Env, the subunit 1 gp120 contains hypervariable loop domains which can mutate quickly in response to antibody pressure. SARS-CoV-2 has also rapidly evolved escape mutations in the subunit 1 receptor binding domain (RBD) and N-Terminal domain (NTD), which has rendered antibody therapies and vaccines less effective. Despite its critical role in receptor binding, viruses can acquire a myriad of mutations in subunit 1 while retaining infectivity (reviewed by (30, 31)). Class 1 fusion proteins are additionally covered with glycans to shield the core proteins from antibody detection (32, 33). Glycans are strategically positioned along key sites of vulnerability and conserved domains. Although antibodies can be made to either penetrate glycan shields or bind directly to glycans, viruses readily mutate glycosylation sites on these fusion proteins. Mutating these glycosylation sites compounds the structural heterogeneity and immune evasive plasticity. More conserved domains of subunit 1, such as the inner-domain of the SARS-CoV-2 Spike RBD and the cluster A epitope of HIV Env, are conformationally occluded and transiently exposed for/upon receptor binding.

Subunit 1 balances viral fitness and plasticity while acquiring mutations to evade immune pressure. By contrast, subunit 2 is more highly conserved and less tolerant of mutations (34–36). This is likely due to the conserved nature of the subunit 2 extension and refolding processes leading to membrane fusion. This apparent conservation suggests that subunit 2 should be an ideal target for broadly anti-viral protection. However, viruses have evolved multiple strategies to prevent effective immune recognition of this vulnerable fusion machinery. The receptor-binding subunit 1 acts as a protective cap, physically covering subunit 2 in the pre-fusion conformation. This arrangement hides conserved epitopes in subunit 2 and focuses immune responses on the more variable subunit 1. Furthermore, many subunit 2 epitopes are only exposed transiently during the prefusion-to-postfusion transition. Because these conformational intermediates exist only for short periods of time, antibody recognition is kinetically disfavored. Rare antibodies capable of binding subunit 2 must often approach from uncommon angles or wait for partial opening of the trimer (37, 38). Several neutralizing sites in subunit 2 lie in close proximity to the viral envelope, where antibody binding is restricted in this sterically crowded environment. In HIV gp41, antibodies that can reach the membrane-proximal external region (MPER) often use unusually long hydrophobic loops to reach the base of the glycoprotein (37, 39–41). The location of subunit 2 refolding may also modulate binding of subunit 2 targeting antibodies. Viruses may fuse in endosomal compartments rather than at the plasma membrane, thereby preventing exposure of the subunit 2 to antibodies in the extracellular space.

#### HIV-1 Env mediates fusion

During biosynthesis, furin cleaves HIV-1 Env gp160 precursor into the gp120 and gp41 subunits, which are held together noncovalently in a meta-stable state (42). While the gp120 subunit mediates engagement of host receptors CD4 and the G-protein coupled co-receptors CCR5 or CXCR4, the gp41 subunit mediates fusion of the virus and host cell membranes. HIV-1 Env can spontaneously adopt multiple conformational states in its unliganded form which are commonly referred to as state 1 (closed), state 2 (partially open), and state 3 (fully open). The presence of CD4 receptor induces the open conformation of Env and antibodies or other host factors may induce a variety of different conformational states (43–46). During viral entry, HIV-1 Env first binds to CD4 on the cell surface of host cells asymmetrically engaging one, two, and three CD4 molecules (10, 47–51). Following CD4 engagement, a large conformational change involving the formation of the “bridging sheet” causes the rearrangement of the V1/V2 loops from the trimer apex outwards aligning parallelly to CD4, and exposing the co-receptor binding site and V3 loop (52, 53). This structure is what is known as the fully open conformational state. HIV-1 Env then engages either the CCR5 or CXCR4 co-receptor—both G-protein coupled receptors with several transmembrane spanning domains (54–60). The structure of the CCR5 co-receptor in complex with HIV-1 gp120 and soluble CD4 revealed that the V3 loop of gp120 extends into the chemokine recognition site 2 (CRS2) of CCR5, and that the N-terminus of CCR5 establishes additional contacts with CD4-induced bridging sheet of gp120 (55). HIV-1 Env primarily utilizes CCR5 co-receptor during transmission and early in infection. Later in infection HIV-1 Env can evolve to utilize CXCR4 as co-receptor (61, 62).

The HIV-1 gp120 and gp41 subunits of Env are separated by a furin cleavage sequence. Immediately downstream of this furin cleavage site on gp41 lies the N-terminal HIV-1 Env fusion peptide. The fusion peptides are highly dynamic and have been shown to be either partially sequestered or exposed in the unliganded prefusion trimer (48, 53, 63, 64). The fusion peptide conformation changes in response to CD4 binding and is coupled to Env trimer opening (48, 53, 64–66). Downstream of the HIV-1 fusion peptide lies the HR-N domain, which is comprised of alpha-helical domains separated by a disordered loop region in the prefusion Env (15). Upon binding to receptor and co-receptor, this disordered loop is believed to begin a loop-to-helix transition, which extends the HR-N and fusion peptide towards the host membrane (15, 48). The fusion peptides are then believed to extend into the host membrane to form the prehairpin intermediate structure, which bridges the virus and host cell-membranes. It is not well understood how or where gp120 moves to allow for extension of gp41. The prehairpin intermediate structure subsequently refolds through interactions between HR-N and HR-C. The coiled-coil domains of HR-N and HR-C regions then transition into a trimeric 6-helix bundle, which pulls the virus and host membranes together and forms the postfusion conformation of HIV-1 Env (15, 67). The membrane proximal external region (MPER) and the fusion peptide proximal region (FPPR) also form interactions during refolding which may induce viral membrane curvature and facilitate fusion (67). Small peptide inhibitors which bind to the prehairpin intermediate were used to capture these structures by electron tomography both on the surfaces of cell-lines and *in vivo* (27, 28).

Recent data suggests that multiple Env trimers may cooperatively orchestrate the membrane fusion process. CryoET analysis of Env-CD4 interactions in membranes revealed how Env forms clusters at membrane-membrane interfaces, which spread into a ring-like pattern as membranes approach (47). Env clustering at membrane-membrane interfaces observed by cryoET was reduced on immature virions (47), consistent with super-resolution microscopy showing that Env surface mobility and clustering are higher on mature than on immature virions (68, 69). This difference is dependent on the Env cytoplasmic tail, which interacts with the matrix protein of the Gag polyprotein precursor that forms a lattice underneath the inner viral membrane leaflet (70). It was also shown that matrix maturation corresponds to faster viral fusion kinetics (71). Together, these data point towards a model where the immature matrix layer restricts Env mobility on the viral membrane, whereas virion maturation releases this constraint, allowing Env to cluster and form fusion interfaces. While Env trimers likely act cooperatively during fusion, it is unclear exactly how many trimers are required for fusion to occur (reviewed by (6)). The number of Env trimers required for fusion has been estimated to be 1–7 trimers per virion, which varies depending on the strain (72). These numbers were mathematically derived using infectivity data from viruses produced with titrated dominant negative, fusion deficient Env. Infectivity of different HIV-1 strains negatively correlated with the number of trimers required for fusion (72, 73). Super resolution microscopy combined with single virus tracking were used to probe the oligomeric states of receptor molecules bound to viruses on living cells (56). These experiments revealed that a CCR5-tropic HIV-1 strain required 1 trimer to mediate entry, whereas a CXCR4-tropic strain required at least two trimers (56).

#### Coronavirus spike mediates fusion

Human coronaviruses vary widely in their receptor usage and often take advantage of transmembrane proteases and/or sialoglycan receptors to mediate entry. SARS-CoV-2, SARS-CoV, Human Coronavirus NL63, (HCoV-NL63) use host receptor angiotensin converting enzyme 2 (ACE2) to mediate attachment (74–76). Other coronaviruses such as Human Coronavirus-229E (HCoV-229E) and Middle East Respiratory Syndrome-related virus (MERS-CoV) bind to human aminopeptidase N (APN) and dipeptidyl peptidase-4 (DPP4), respectively (77–80). Recently, it was shown that HKU1 utilizes transmembrane serine protease (TMPRSS2) as a receptor (81–84). In addition to proteinaceous receptors, coronaviruses such as SARS-CoV-2, MERS-CoV, HKU1, and recently discovered CCoV-HuPn-2018, also interact with sialoglycans to facilitate entry (84–89). Other coronaviruses such as OC43 are only known to interact with sialoglycans as receptors but may require additional factors to induce the conformational changes needed for entry (90).

The Spike proteins from coronaviruses exhibit different conformational states of their receptor-binding domains (RBDs) on the S1 subunit, including “up” and “down” conformations (91–93). Spike transitions to the “up” conformation to bind the host receptors, and the “down” conformation to potentially hide conserved epitopes from immune surveillance (91–94). Transitions between RBD-up and RBD-down conformations of the SARS-CoV-2 Spike occur spontaneously, but these conformational states can be stabilized by ligand binding (94–96). For HKU1 and SARS-CoV-2, binding to sialoglycans can induce the RBD-up conformation, which may add further regulation to prevent premature exposure of immunogenic epitopes (84, 97). Single-molecule Förster Resonance Energy Transfer (smFRET) has been used to probe how the conformational dynamics of SARS-CoV-2 Spike change in response to ligands such as soluble receptor and antibodies, pH, and calcium concentrations in real-time (94–98). Binding of ACE2 stabilizes the SARS-CoV-2 “up” conformation, whereas some antibodies can stabilize “up” or “down” conformational states (94). Lower pH facilitates RBD-up transitions (98), whereas low pH and Ca^2+^ promotes the S2 subunit refolding in later fusion events (95, 99). Analyses of Spikes from SARS-CoV-2 variants of concern (VOCs) revealed how Spikes acquired mutations (i.e. D614G and E484K) which stabilize RBD-up conformations (100). The real-time conformational landscape of coronavirus Spike RBDs besides SARS-CoV-2 Spike are largely unknown and remain an active area of research.

In addition to RBD-up/down transitions, the inherent flexibility of both Spike and receptor molecules may facilitate entry. Coronavirus Spikes have a long flexible stem region that is generally not well-resolved and often truncated to produce soluble spike ectodomains for determining high resolution structures with single-particle cryoEM (74, 92, 101, 102). However, the high-resolution structure of full-length Spike on virions was also solved with single-particle cryoEM (93). CryoET studies of Spikes on membranes have revealed extensive tilting through three flexible hinges known as the “hip”, “knee”, and “ankle” (93, 103, 104). CryoET analysis of native HCoV-NL63 virions demonstrated how Spike tilting is conserved among diverse coronaviruses and that glycans near the hinge regions may modulate tilting and immune evasion (105). ACE2 receptor is also known to be quite flexible on the membrane, as shown through all-atom based molecular dynamics simulations and cryoET analysis (18, 106). Both Spike and receptor having high inherent flexibility likely facilitates viral attachment.

Spike and receptor flexibility may also play a role in receptor-induced cross-linking at membrane-membrane interfaces. Recently, it was shown using cryo-electron tomography that ACE2 dimers could cross-link SARS-CoV-2 Spikes in membranes (18). ACE2 dimers were not observed to bind to two RBDs on the same Spike molecule. Rather, ACE2 could only bind to one RBD on the same Spike, which leaves the other end of the ACE2 dimer free to cross-link other Spikes (18). Other known coronavirus receptors such as DPP4 and APN are also dimeric (79, 107, 108). Whether MERS-CoV and/or HCoV-229E Spike cross-linking occurs through DPP4 and APN, respectively, is an active area of research. Structural modeling predicts that DPP4 could cluster MERS-CoV Spikes (91). How receptor-mediated cross-linking of Spikes affects the viral fusion process remains unclear; it is possible that clustering of multiple Spikes may collectively orchestrate the fusion process for these coronaviruses.

Like HIV-1 Env, coronavirus Spike proteins are cleaved by cellular proteases into subunit 1 and subunit 2 (S1 and S2) (74, 109–112). Some coronaviruses, such as SARS-CoV, have less optimal cleavage sites which may not become processed until reaching target cell proteases (110, 111). It has been demonstrated that the polybasic furin cleavage site is required for SARS-CoV-2 transmission in ferrets and facilitates replication in human airway epithelial cells (113). The effect of the S1/S2 cleavage sequence on SARS-CoV-2 and MERS-CoV cellular entry is cell-type dependent, with human lung cells expressing TMPRSS2 (i.e. Calu3 cells and human airway epithelial cultures) generally less susceptible to infection with S1/S2 cleavage site mutants (113, 114). Unlike HIV-1 Env, coronaviruses Spike proteins undergo a second proteolytic processing step at the S2’ site, which lies ~50 residues upstream of the fusion peptide (17, 112, 115, 116). The S2’ site is conformationally exposed upon ACE2 binding (for SARS-CoV-2) or DPP4 binding (for MERS-CoV) and may be processed by cell-surface proteases, such as TMPRSS2, or by endosomal cathepsins (114, 117, 118). Cleavage at the S2’ site facilitates membrane fusion and viral entry (119), although the molecular details of how S2’ cleavage affects membrane fusion remains unclear.

After attachment to host receptor and the S1/S2 proteolytic processing, the S1 subunit displaces away to allow for the extension (loop to helix transition) of the S2 subunit and insertion of the fusion peptides into the host membrane. The Spike which bridges the virus and host membranes then refolds to pull the two membranes together. This activation and refolding of Spike was modeled by previous molecular dynamics simulations (26). The HR-N domains from the three protomers undergo a loop-to-helix transition to extend the fusion peptides towards the host membrane, forming the “prehairpin intermediate” as an extended, continuous trimeric coiled-coil comprised of the central helix (CH) domain and the HR-N domain (26, 120). During this extension, the S2 stem coiled-coil domain separates to accommodate motion of the “head-group” towards the viral membrane (26). The CH/HR-N and the HR-C/upstream linker domain then associate in a “zippering” like fashion, thereby pulling the virus and host membranes together to form the postfusion 6-helix bundle (17, 26, 120).

CryoEM/cryoET studies have significantly improved our mechanistic understanding of Spike activation and refolding. CryoET studies using coronavirus virus-like-particles (CoV-2 VLPs) or HIV-1 pseudoviruses decorated with Spike were able to capture the unstable and highly dynamic S2 intermediate conformations in membranes (18, 25). In the former, CoV-2 VLPs were mixed with ACE2 extracellular vesicles in the presence of a lipopeptide fusion inhibitor. CryoET images showed the prehairpin intermediate in various conformations from extended intermediates to nearly completely refolded intermediates with contacting membranes (25). The later study used a dual virus-like particle approach, co-incubating HIV-1 virus decorated with spike and murine leukemia virus decorated with ACE2 (18). This increased the frequency of observable events to facilitate subtomogram averaging. The low-resolution structures of prehairpin intermediates from subtomogram averaging corresponded well with models of Spike refolding derived from previous molecular dynamics simulations (18, 26). A subsequent study used authentic coronaviruses mixed with ACE2_VLP_s and demonstrated that the addition of trypsin facilitates fusion between the viruses (119). The final postfusion structure of SARS-CoV-2 Spike was solved which includes the structures within the membrane (17). This structure revealed how the three fusion peptides each adopt a hairpin-like structure spanning the membrane, and the transmembrane domains are positioned around the fusion peptides (17).

In addition to these cryoET studies in opposing membranes, single particle cryoEM studies have provided a detailed understanding of these S2 intermediate structures. A single particle cryoEM analysis of the extended S2 subunit with ACE2-bound S1 subunits still non-covalently attached (116). These S1-ACE2 complexes subsequently shed entirely during Spike refolding and remain intact on membranes, which may have unknown physiological implications (18). The high-resolution structure of the extended S2 subunit revealed that upon ACE2 binding, the S2’ site becomes fully exposed near the host membrane and lies along the extended HR-N domain (116). Thus, the S2’ site is likely cleaved after extension of the prehairpin intermediate and insertion into the target membrane. It is possible that residues 769–831 which lie along the extended HR1 domain play a role in immune evasion or modulate subsequent associations with the HR2 domain during refolding (116). S2’ cleavage does not appear to be necessary for the extension and insertion of fusion peptides into the host membrane or Spike refolding into the postfusion conformation. Single particle cryoEM analysis revealed Spikes transitioning from the prefusion to postfusion conformation in the presence of ACE2 alone (17). Further, cryoET studies have demonstrated that extended S2 subunits inserted into ACE2-bearing membranes can form in the absence of protease (18). However, S2’ cleavage facilitates membrane fusion after insertion of the fusion peptides (119). The location of S2’ proteolytic processing may partially determine whether Spike-mediated entry occurs at the plasma membrane or within endosomes (121).

Low pH and calcium concentration have been implicated in the conformational dynamics of SARS-CoV-2 refolding. First, higher pH has been shown to inhibit SARS-CoV-2 entry, particularly in cell types which do not express TMPRSS2, likely because cathepsin L necessary for cleavage is activated at lower pH (121). Lipid mixing assays with Spike VLPs and liposomes have shown that SARS-CoV-2 and MERS CoV Spike-mediated fusion is facilitated by both calcium and low pH (99, 122). Single molecule FRET experiments using S2 protein tagged with fluorophores also demonstrated that S2 conformational changes are stimulated by pH and calcium (95, 99, 122). Interestingly, the S2 conformational changes promoted by low pH and calcium are reversible in the absence of a target membrane pointing to an important role of the membrane in promoting conformational changes associated with fusion (95, 122).

## Neutralization via binding to viral fusion proteins

**Figure 2:**
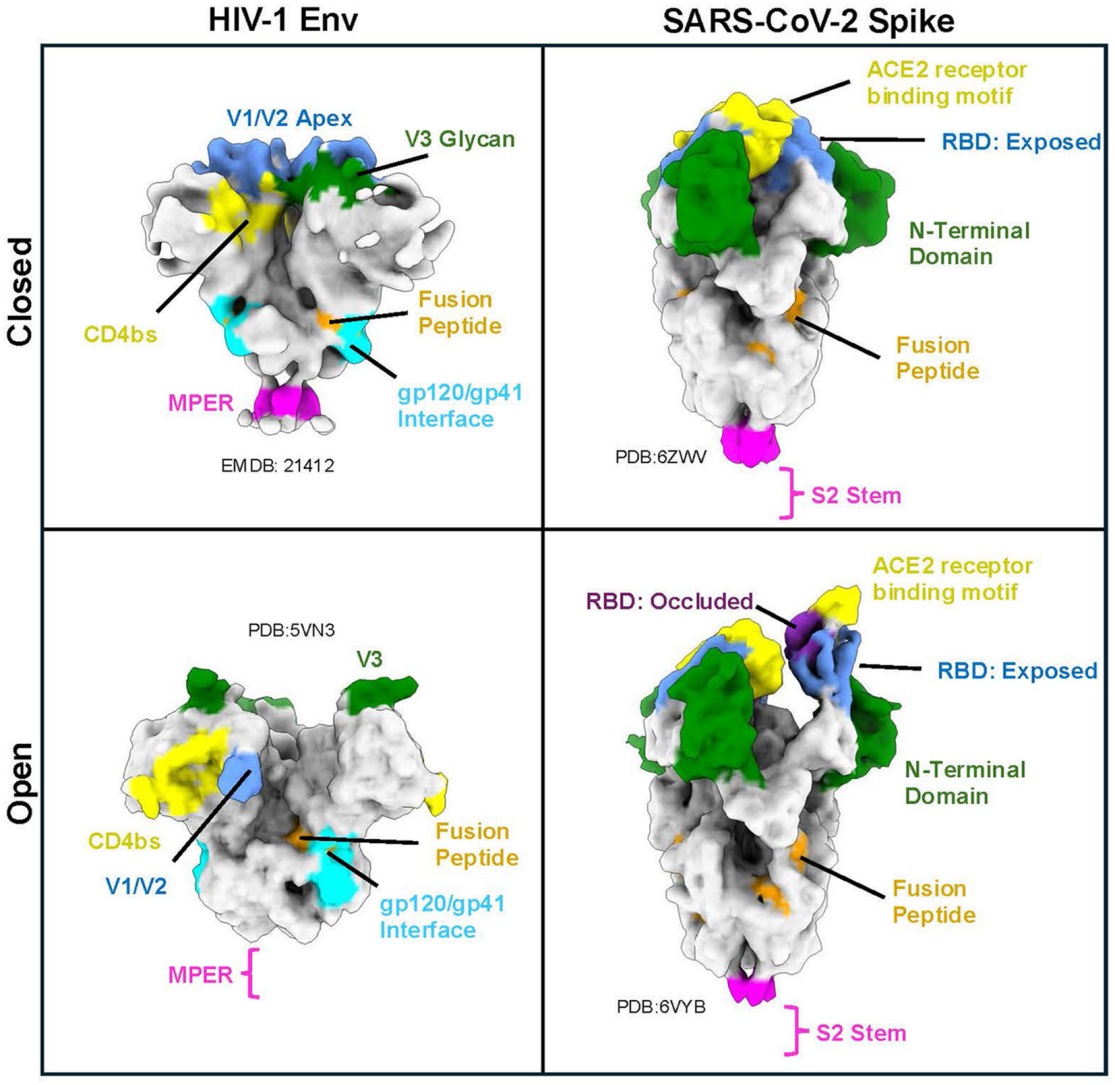
Major epitopes on viral glycoproteins of HIV-1 and SARS-CoV-2 targeted by neutralizing antibodies (53, 74, 93, 123–125). Top: closed conformations of the viral fusion glycoproteins. Bottom: receptor-bound open conformations. Left: HIV-1 Env (CD4bs: CD4-binding site, MPER: membrane proximal external region), Right: SARS-CoV-2 Spike (RBD: receptor binding domain). “RBD: Occluded” includes residues not accessible to antibodies in the RBD-down conformation, whereas “RBD: Exposed” includes residues accessible in both the RBD-up and RBD-down conformations. Indicated PDBs were converted to density maps at 9 Å resolution in ChimeraX for simplified visualization (126, 127).

**Figure 3:**
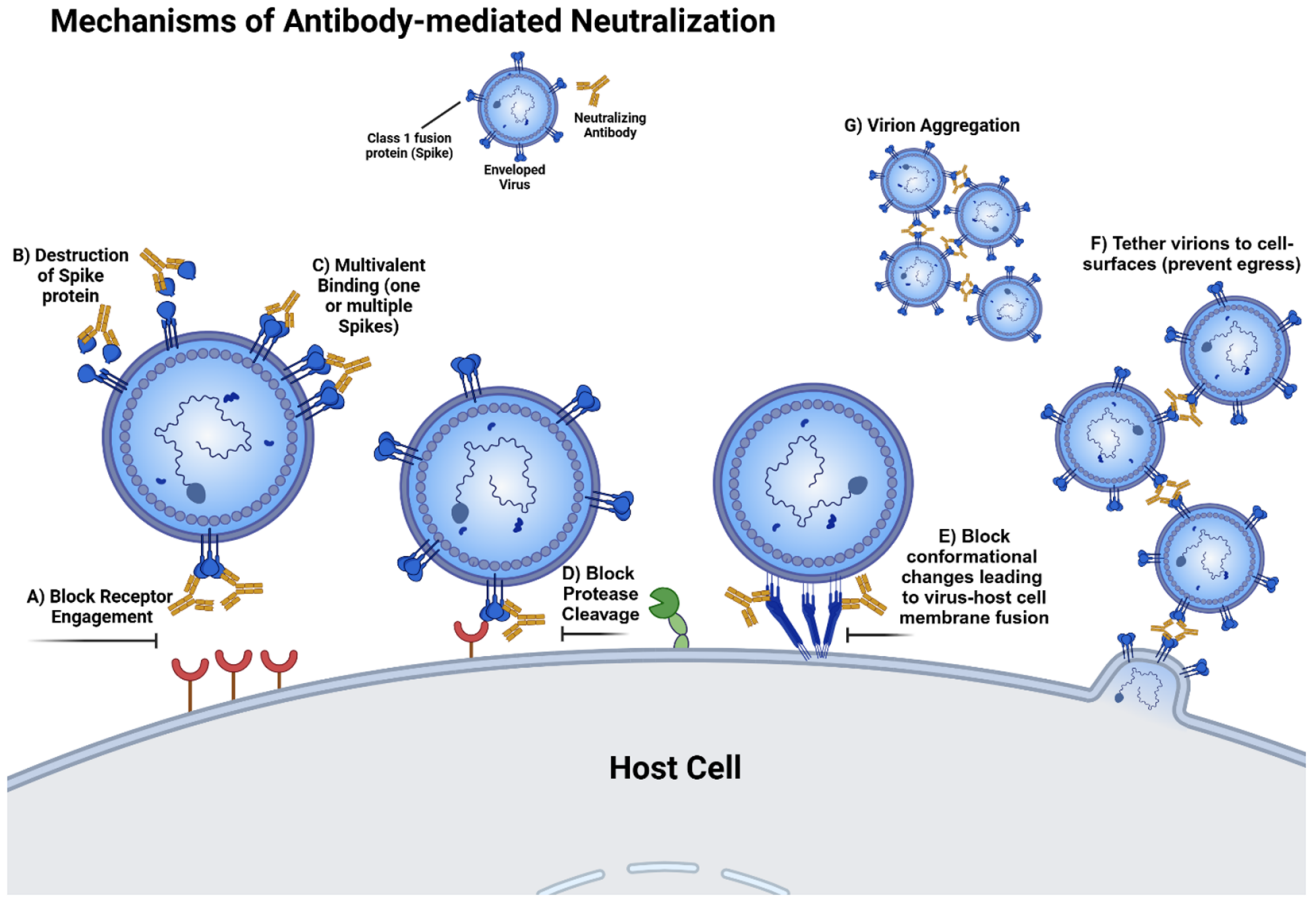
Mechanisms of Antibody-mediated neutralization. **(A)** There are several ways in which antibodies can interfere with attachment to the host receptors, including receptor mimicry (competitive inhibition), allosteric (stabilization of prefusion conformation, and steric blocking. **(B)** Antibodies may trigger viral fusion glycoproteins prematurely and dismantle them. **(C)** Divalency allows some antibodies to bind multiple protomers on one trimer or multiple protomers from different trimers. **(D)** Antibodies can block the protease cleavage site on viral fusion proteins requiring cleavage after biosynthesis (i.e. coronavirus Spike proteins). **(E)** Antibodies may bind to different conformations during activation and refolding, thereby blocking the transition into the postfusion conformation and preventing virus-host membrane fusion. **(F)** Antibodies can bind cell-surface viral fusion proteins and budding virions simultaneously which prevents viral egress. **(G)** Antibodies can aggregate virions by binding to multiple trimers from different virions. Note that some of these functions (i.e. blocking receptor engagement and protease cleavage) may occur before or after viral endocytosis and may also prevent cell-cell fusion. This figure was created in BioRender. Uchil, P. (2026) https://BioRender.com/v00u103.

Class 1 viral fusion glycoproteins are among the most prominent antigens on the surface of virions, making them the primary target of the immune system and vaccine design. There are multiple sites of vulnerability on viral fusion glycoproteins which have been exploited for generating vaccines and monoclonal antibodies (reviewed by (123, 124)) (**Figure 2**). “Neutralization” of viral infectivity typically refers to antibodies that, besides binding to viral fusion glycoproteins on virions, also prevent the establishment of infection in host cells. This phenomenon is observed in common virus neutralization assays, in which antibodies are preincubated with virions before the addition to cells and the amount of viral entry or infection is assessed afterwards. While these assays quickly measure the ability of antibodies to neutralize infectivity, they do not provide mechanistic detail. Recent insights have shown the potential mechanisms of neutralization to be more diverse and complex (reviewed by (128)) (**Figure 3**). Antibodies may block interactions with receptor through competitive binding, steric hindrance, or allosteric inhibition of conformational changes necessary for receptor binding. Antibodies can also cause premature inactivation of viral fusion glycoproteins (receptor mimicry) or block the fusion process after attachment. Further, antibody multivalency may contribute to neutralization through increasing avidity and/or cross-linking multiple viral fusion proteins together.

### Antibodies against subunit 1

While antibodies targeting subunit 1 neutralize through a variety of mechanisms, many of these antibodies directly block interaction with host receptors through competitive inhibition or steric inhibition. For HIV-1, gp120-targeting antibodies to the CD4-binding site (CD4bs) directly compete with CD4 receptor binding. Other antibodies targeting the V3 glycan, and V1/V2 apex may also prevent engagement with co-receptors CCR5 or CXCR4. Given the relatively small size of HIV-1 Env (3 x 160 kDa protomers) compared to an antibody (~150 kDa), it seems intuitive that most antibodies capable of binding to prefusion closed HIV-1 Env gp120 with enough affinity can inhibit attachment and/or membrane fusion either by direct receptor blocking or steric inhibition. In addition to 1) competitive inhibition with binding to receptor, other mechanisms of neutralization include: 2) allosteric inhibition, e.g. antibodies stabilizing the prefusion conformation allosterically inhibiting the adaption of receptor-bound states, and 3) premature activation in the absence of target membranes that leads to inactivation (44, 94, 129–132).

CD4 binding site antibodies to HIV-1 Env, such as 3BNC117, VRC01 and N6, can exhibit broad and potent neutralization through partial CD4 receptor mimicry (133–136). Passive transfer of 3BNC117 antibody was shown to suppress viremia in people living with HIV-1 (PLWH) (137), and also delayed viral rebound after anti-retroviral therapy (ART) interruption (138). Passive transfer of VRC01 antibody to individuals at-risk of HIV-1 infection was associated with lower acquisition of VRC01 sensitive strains and lower viral loads at time of detection, although it did not prevent viral acquisition overall (139). VRC01 was shown to enhance binding of antibody 17b, which binds to the co-receptor binding site (CoRBS). The CoRBS is normally occluded on the prefusion unliganded trimer and becomes exposed upon CD4 binding. This data suggests that VRC01 induces at least a partial conformational opening of the trimer (134, 140).

Antibodies to the V1/V2 apex have also been a major focus in HIV-1 viral immunology. The V1/V2 apex contains a relatively conserved cationic domain across HIV-1 strains as well as SIV, although in both cases it is occluded by glycans and surrounded by variable loops (141, 142). PGT145 and other similar antibodies recognize a quaternary epitope at the Env trimer 3-fold axis of symmetry comprised of the cationic domain. One V1/V2 apex antibody, PGT145, binds in this conserved cationic domain and can cross-react with SIV Env (143). A long complementary determining region (CDRH3) of PGT145 and other similar antibodies contains sulfated tyrosines which penetrate the glycan shield Env by reaching into this 3-fold axis (142, 144, 145). V1/V2 apex glycans are also part of this epitope, and loss of the V1/V2 apex glycans in both HIV-1 Env and SIV substantially reduces PGT145 binding (142–146). SIV bearing a K180S mutation in Env (to increase neutralization sensitivity) acquired resistance to passively transferred PGT145 antibody by mutating the V1/V2 apex glycans, highlighting their importance for recognition (147). Although PGT145 does not directly interfere with CD4 binding, it may sterically prevent membrane-bound CD4 from reaching the binding site, as well as prevent CoRBS engagement with co-receptor (144). In agreement with structural data, single molecule FRET analysis suggests that PGT145 and other V1/V2 apex antibodies stabilize a closed conformation hindering the opening induced by receptor CD4 (43, 44).

The SARS-CoV-2 S1 receptor-binding domain (RBD) has been a major focus in the development of vaccines and neutralizing antibodies to block engagement with ACE2 receptor. In fact, immunogenic sites on the Spike RBD have been subcategorized extensively (102, 148), (reviewed by (123, 149)). Antibodies which target different faces of the RBD have diverse properties. Antibodies can bind and stabilize the RBD-up conformation, but their epitopes may lie inside or outside the ACE2 binding site (94, 102, 148). Others have been shown to bind and stabilize RBD in the “down” conformation thereby allosterically preventing receptor binding (94, 102, 150). Some antibodies can bind both the RBD-up or RBD-down states (102). Multiple Fab arms from a single antibody can either engage two RBDs on the same Spike or from different Spikes (see more on antibody multivalent binding below) (148, 151). Because the RBD is one of the most immuno-dominant epitopes of the SARS-CoV-2 Spike, variants of concern (VOC) heavily mutate this region to evade the presence of antibodies in the human population (reviewed by (31)). Interestingly, the most conserved epitope is the region where the inter-protomer RBD-RBD interfaces in the RBD-down conformation (**Figure 1B**, RBD: Occluded) (102, 152–154). Consequently, it is this epitope that has been a focus for vaccine approaches (155, 156).

While directly competing for or sterically blocking receptor binding is the inhibitory mechanism of many potently neutralizing antibodies, there are other ways in which antibodies binding to subunit 1 can inhibit infection. Anti-HIV-1 antibodies such as 10–1074 can tether viruses to cell surfaces and prevent viral egress (157). Some antibodies against SARS-CoV-2, such as CV3–1 not only bind the RBD and block the ACE2 interaction but also can induce shedding of S1 and premature triggering of the Spikes into the postfusion state (129–132). Antibodies against the N-Terminal Domain (NTD) of SARS-CoV-2 Spike may also neutralize infectivity in distinct ways. Binding of antibodies to a quaternary epitope of NTD-subdomain 1 interface might affect ACE2 binding by locking RBD in a down conformation (158). Alternatively, NTD antibodies may not directly block spike binding to ACE2 protein (159, 160). However, removal of the Fc domain (Fab_2_ fragments) can reduce their neutralization potency, suggesting that the antibody Fc domain may sterically hinder the RBD-ACE2 interaction in membranes (158, 159). Fab fragments have also been shown to completely lose neutralization potency compared to Fab_2_ fragments which leaves open the possibilities of avidity and/or Spike cross-linking being critical for neutralization (160).

### Antibodies against subunit 2

Developing and eliciting antibodies against subunit 2 (HIV-1 gp41, SARS-CoV-2 S2) is of interest to the field of vaccinology and viral immunology. As subunit 2 contains the hard-wired viral fusion machine, epitopes on subunit 2 are more conserved (36, 161) and antibodies binding to these epitopes can provide broad immunity. Unlike antibodies to subunit 1, antibodies to subunit 2 often do not prevent glycoproteins binding to host receptors and can neutralize downstream of host receptor engagement (18, 39, 162) (reviewed by (163, 164)). Some subunit 2 antibodies bind to epitopes only transiently exposed upon Env/Spike refolding during the membrane fusion process. Consequently, subunit 2 antibodies often feature reduced neutralization potency compared to subunit 1 antibodies. Despite this, several subunit 2 antibodies have been shown to mediate protection against infection.

Antibodies to the HIV-1 Env gp41 domain are some of the most broadly neutralizing (39, 124, 165). Two main sites of vulnerability on the gp41 domain are the membrane-proximal external region (MPER) and the fusion peptide. The MPER is located at the base of the trimer, is partially embedded in the lipid membrane and the epitope is more fully exposed as the antibody binds and the Env tilts (37, 70, 166). The epitope is also believed to be more accessible to antibodies upon CD4 binding (162). Antibodies which bind to the MPER often also interact with viral membrane lipids (167) (reviewed by (163)). Some antibodies against the MPER, such as 2F5 and 4E10, react strongly with self-antigens such as host cardiolipin (168). A clinical study showed that levels of anti-cardiolipin antibodies are associated with higher viral loads and proviral DNA in people living with HIV-1 (PLWH) (169). This study also demonstrated that higher levels of 2F5 and 4E10-like antibodies are associated with higher levels of anti-cardiolipin antibodies (169). It is hypothesized that the induction of antibodies targeting the MPER are limited due to immune tolerance mechanisms (168). Supporting this hypothesis, expression of 2F5 and 4E10 antibody sequences in knock-in mice was shown to induce immune tolerance mechanisms that inhibited B cell development (170–172). While autoreactivity is a concern in vaccine development, other antibodies to the MPER region have been isolated which are less polyreactive, such as PZGL1, HK43, 10E8, and DH511 (39–41). Recent human vaccine trials using MPER peptide-liposome immunization have shown that heterologous neutralizing antibodies to the MPER can be elicited without inducing high autoimmunity (173).

Antibodies to the MPER neutralize by distinct mechanisms to other Env-targeting antibodies. Post-attachment neutralization assays and antibody wash-out assays suggest that MPER antibodies may not bind well to prefusion Env on virions, binding is increased upon CD4 receptor engagement, and they neutralize after virus attachment to cells (39, 41). It was also demonstrated that gp41-inter proteins (mimicking the gp41 prehairpin intermediate) can block 4E10 neutralization even when the antibody is pre-incubated with the virus (167). These results support a model that 4E10 binds transiently/reversibly to viral membranes to increase local concentrations on the virion. Upon triggering of HIV-1 Env, the MPER epitope becomes exposed and the antibody binds irreversibly, effectively neutralizing through blocking the gp41 transition to the postfusion state (163, 164, 167).

The fusion peptide is another major focus in HIV-1 viral immunology. The fusion peptide is comprised of hydrophobic residues near the gp41 N-terminus. This peptide is highly dynamic and changes conformation in response to ligands (see above). In a way, we can view the fusion peptide as the region of Env that is constantly searching for the presence of the target membrane. The fusion peptide can be captured by broadly neutralizing antibodies on the prefusion unliganded Env trimer and is therefore a major focus in the development of vaccines (174–179), (reviewed in (180)). Broadly neutralizing antibody VRC34.01 is a fusion peptide-targeting antibody isolated from a participant with a chronic HIV-1 infection and exhibits broad and potent neutralization properties (175). Unlike MPER antibodies, VRC34.01 neutralizes in an antibody washout assay which suggests that it targets a prefusion epitope readily accessible on virions (175). VRC34.01 does not inhibit CD4 binding but prevents refolding of the bridging sheet as assessed by binding of co-receptor antibody, 17b (175). VRC34.01 only recognizes cleaved Env trimer, and smFRET analysis indicates that VRC34.01 stabilizes a partially open conformation (175). Together, these results suggested that VRC34.01 neutralizes by engaging cleaved, prefusion trimers and stabilizing a partially open intermediate state of Env. Although likely, whether these same detailed mechanisms extend to other fusion-peptide targeting antibodies is not well-known.

The SARS-CoV-2 Spike S2 subunit may represent a valuable component of vaccines to elicit cross-reactive antibody responses and protect against emerging coronaviruses (reviewed by (35, 181)). The S1 subunit has acquired a myriad of mutations rendering monoclonal antibody therapies ineffective and reducing vaccine efficacy (182–187), whereas the S2 subunit has remained relatively conserved. Several antibodies against S2 can cross-react with diverse coronaviruses, such as SARS-CoV and MERS-CoV (reviewed by (149)). The S2 subunit contains immunogenic epitopes that elicit broadly-reactive antibody responses upon vaccination or infection (188–191). Some of these antibodies are thought to derive from B cells previously activated by common cold coronaviruses (188, 191, 192). Several anti-S2 antibodies targeting distinct regions have been characterized (reviewed by (181)) with distinct neutralization mechanisms. The two major known sites of vulnerability of the Spike S2 subunit are the stem-helix region and the fusion peptide (149), although some antibodies to other domains such as the HR1 region and S2 apex region have been described (193–195).

The first group of anti-S2 antibodies target the Spike coiled-coil stem-helix region just upstream of the HR2 domain (129, 196–202). Most of these antibodies bind to the buried, more conserved hydrophobic face of the stem-helix, and their epitopes are seemingly not exposed on prefusion Spikes (196–201). However, they can readily bind prefusion Spikes on membranes, suggesting that the coiled coil domain must “breathe” to allow for binding (18). A cryoEM structure of stem-helix antibody S2P6 bound to SARS-CoV-2 Spike ectodomain suggests the coiled-coil quaternary epitope is disrupted to allow for antibody access (199). The stem-helix is seemingly protected by the N1158 glycan, although mutagenesis of this glycosylation site has limited impact on neutralization for at least four antibodies (196). The second type of stem-helix antibody targets the exposed face of the stem-helix. The only known antibody of this category is CV3-25, which was isolated from a convalescent patient (129, 202, 203). CV3–25 is generally more potent than antibodies targeting the inner face of the stem-helix, although it does not cross-react with MERS-CoV (196). Kapingidza et al. engineered the Spike stem-helix region onto scaffolded immunogens and found effective protection when used as a booster in Spike mRNA pre-vaccinated mice (204). These focused immunization strategies may serve to elicit broadly reactive immune responses to the stem-helix region (204).

S2 stem-helix antibodies mechanistically neutralize by preventing refolding of S2 during membrane fusion and do not prevent binding to ACE2 receptor (18). Dual virus-like particles carrying Spike or ACE2 receptor were used to probe the neutralization mechanism of anti-S2 antibodies at membrane-membrane interfaces (18). CV3–25 was shown to bind to prefusion Spike-ACE2 complexes in membranes and prevent Spike tilting (18). Upon spike activation, CV3–25 remained bound to prehairpin intermediate Spikes in membranes, which prevented the two viral membranes from approaching (18). Molecular dynamics simulations further demonstrated how CV3–25 could prevent Spike refolding and provided molecular details how binding to the epitope prevents back-zippering of HR-2 along HR-1 (18, 26). In addition to CV3-25, two other antibodies targeting the inner-face of the stem-helix, CC25.106 and CC99.103, also bound to prehairpin intermediates and increased distances between Spike and receptor membranes during Spike refolding (18).

The second major site of vulnerability on the Spike S2 domain is the fusion peptide. Highly cross-reactive antibodies to the fusion peptide have been isolated, some of which have been shown to neutralize all seven coronaviruses known to infect humans (205–207). Antibodies to the SARS-CoV-2 fusion peptide including 76E1, VN01H1, and C77G12 were shown to bind upon Spike-ACE2 engagement or engagement with RBD-targeting antibodies, indicating that the epitope may be conformationally masked in the unliganded Spike (206, 207). Fusion peptide antibodies were shown to neutralize SARS-CoV-2 Spike S2 function by occluding the S2’ site (which is exposed by ACE2 binding) and preventing subsequent TMPRSS2-mediated S2’ cleavage (206). VN01H1 and C77G12 were unable to bind SARS-CoV-2 ACE2-activated Spikes containing prefusion stabilizing 2P mutations found in the mRNA vaccines, perhaps indicating that current mRNA vaccines may be suboptimal at inducing these types of antibodies (207). Supporting this hypothesis, studies have suggested that COVID-19 infection elicits greater fusion peptide reactivity in sera than vaccination alone (205, 208). Therefore, vaccination strategies including an S2 domain capable of sampling conformations more like the native Spike, along with the prefusion-stabilized Spike, may improve anti-S2 responses.

### Antibody multivalent binding can contribute to neutralization

Antibody multivalent binding to viral fusion proteins also plays a role in neutralization. Antibodies may bind multiple epitopes on different protomers of a single trimeric fusion protein (intra-Spike/Env binding), thereby enhancing their binding through avidity (132, 148). For SARS-CoV-2, this mode of multivalent binding contributes to neutralization potency and can enhance triggering of Spikes into the postfusion state (132). For HIV-1 Env, it was shown through using Fab domains connected via DNA-linker sequences of different lengths that intra-Env binding enhances neutralization potency (209). The ability of both antibody Fab domains to bind multiple protomers on a single trimeric fusion protein is highly dependent on the antigen binding site.

Antibodies may bind multiple different fusion proteins (inter-Env/Spike cross-linking) either on a single virion (*in cis*) or from different virions (*in trans*). HIV-1 Env has evolved to limit Env on virions to approximately 7–14 trimers per virion, which likely reduces inter-Env cross-linking (210, 211). Despite low Env density on virions, some anti-HIV-1 Env antibodies neutralize far more potently in their IgG form than in their Fab form, suggesting Env cross-linking can contribute to neutralization potency (212–214). Additionally, it was shown that some bNAbs can cross-link and tether budding virions to cell surfaces, adding another neutralization mechanism to their overall antiviral activity (157). However, some antibodies targeting different sites on HIV-1 Env, particularly the membrane-proximal external region (MPER), do not show strong improvement in neutralization when comparing IgGs and Fabs (213, 215). These results suggest that the contribution of Env cross-linking to neutralization potency is likely epitope-dependent. To better understand this, Galimidi et al. examined antibodies which bind the HIV-1 Env V1/V2 apex and cannot exhibit intra-spike cross-linking (there is only one V1/V2 apex quaternary epitope per trimer) (209). Using Fab domains connected by DNA linkers, it was shown that longer DNA linkers can enhance neutralization potency of a V1/V2 apex antibody, PG16. However, these enhancements in neutralization potency were modest and observed only on a few HIV-1 strains. Fabs to different sites, such as the CD4-binding site, showed optimal neutralization with linkers likely too short for inter-Env cross-linking, and showed reduced potency with greater linker lengths (209). Altogether, further investigations into how higher order cross-linking contributes to neutralization of HIV-1 Env by antibodies to different epitopes is warranted.

Inter-spike cross-linking has also been observed for anti-SARS-CoV-2 antibodies. Distinct modes of inter-spike cross-linking by IgG were observed with soluble Spikes using cryoEM (148, 151, 216). Soluble Spikes were shown clustered either in a head-to-head trimer-dimer configuration or in an offset configuration (151, 216). An anti-RBD antibody, 6H2, cross-links ancestral Spike in a head-to-head trimer dimer configuration while cross-linking Omicron Spike in offset configuration. The offset configuration is thought to be less potently neutralizing because it may aggregate viral particles at lower efficiency while still allowing partial ACE2 engagement (151). Spike cross-linking has been exploited to enhance neutralization potency of other compounds. An engineered biological platform named adaptive multi-epitope targeting with enhanced avidity (AMETA) was generated which uses 20 or more anti-Spike nanobodies linked together in an IgM scaffold (217). This construct potently neutralizes SARS-CoV-2 infectivity and cross-links Spikes both *in cis* (on a single virion) and *in trans* (linking multiple virions together) (217).

## Antibody Fc effector functions

Although not considered a mechanism of neutralization, antibody Fc effector functions play a critical role in antiviral immunity. Antibodies contain an Fc (crystallizable) fragment domain capable of binding to complement proteins or Fc receptors (FcRs) on immune cells and eliciting Fc effector functions. Antibodies may interact with the C1q subunit of the complement system and initiate a cascade of events that lead to the formation of the membrane attack complex which lyses the target cell (reviewed in (218)). Antibodies can also recruit immune cells bearing Fc receptors to eliminate infected cells and stop the production of new viruses or induce inhibitory signals to dampen the immune response. Fc effector functions often augment the ability of an antibody to mediate protection and eliminate viral reservoirs in cells. Commonly studied cell-mediated Fc effector functions include antibody-dependent cellular cytotoxicity (ADCC), antibody dependent cellular phagocytosis (ADCP), and antibody dependent (virion) phagocytosis (ADP) (reviewed in (219)). There are a variety of Fc receptors on effector cells which react with different immunoglobulins, such as FcɣRs (bind to IgG), FcαRs (bind to IgA), and FcϵRs (bind to IgE) (reviewed in (220)). The most studied FcR-antibody interactions are those between FcɣR and IgG.

Cell-mediated Fc effector functions are triggered through the formation of antigen-antibody-Fc receptor complexes and subsequent Fc receptor signaling. Fc receptors may signal through an intracellular immunoreceptor tyrosine activation/inhibitory motif (ITAM/ITIM) to modulate immune responses (219). These signaling motifs can either be on the same protein as the extracellular Fc binding domain (i.e. FcɣRIIa/b) or on separate signaling subunits such as the common ɣ-chain (i.e. FcɣRI, FcɣRIIIa) (219). The FcɣRIIIb extracellular domain is GPI-anchored and contains no signaling domain (219). Fc receptors on immune cells will bind to various classes or subclasses of antibody Fc domains with different affinity. For example, human FcɣRIIIa preferentially engages human IgG1 and IgG3, and interacts poorly with human IgG2 and IgG4 (221, 222). Different Fc receptor alleles also vary in their interactions with IgG subclasses. IgG2 mediates strong Fc-receptor signaling through the human FcɣRIIa:H131 allele and poorly induces signaling through the FcɣRIIa:R131 allele (221). Immune cells vary in which Fc receptors are expressed, which may modulate their function. For example, natural killer cells (NK cells) which mediate ADCC express FcɣRIIIa. Monocytes may express FcɣRI, FcɣRIIIa, FcɣRIIa, and FcɣRIIb (219). B cells exclusively express the inhibitory FcɣRIIb which modulates the development of the humoral immune response to prevent induction of auto-reactive antibodies (reviewed by (223)).

Recombinant antibodies can be engineered to modulate Fc effector activity (224). The L234A, L235A (LALA) mutations substantially reduce Fc receptor binding, and the addition of a P329G mutation further abrogates the Fc receptor interaction (225, 226). These mutations are useful clinically for dampening unwanted immune responses with antibody treatment, or scientifically for testing the contribution of antibody Fc effector functions *in vivo* (218). Antibodies can also be engineered to enhance Fc effector functions. S239D, I332E, A330L mutations increase binding to FcɣRIIIa, whereas G236A mutations enhance binding to FcɣRIIa (these mutations have also been combined in a “GASDALIE” Fc domain) (221, 224). G236A/A330L/I332E (GAALIE) mutations have been described which increase binding to activating FcɣRs but not to inhibitory FcɣRs (227). Antibody afucosylation also increases affinity for Fc receptors (219) and these antibodies are being tested and used clinically (reviewed in (228)). In the case of SARS-CoV-2 infection, afucosylated antibodies are associated with poorer disease outcomes perhaps through augmented Fc effector functions that drive immunopathology (228).

The ability of an antibody mediate Fc effector functions against virus-infected cells generally corresponds to its ability to neutralize viral infectivity. This is because antibodies capable of binding and neutralizing functional Spike proteins on virions can also bind trimers on infected cells. However, some non-neutralizing antibodies are also capable of mediating Fc effector functions, such as ADCC (229–231). The exact mechanisms of these phenomena are not completely understood. It is possible that there are viral Spike epitopes present on infected cells which are not present on virions (i.e. HIV-1 gp41 postfusion stumps, defective Env, uncleaved Env, or shed gp120) (232–234). For SARS-CoV-2, some ADCC-mediating antibodies, such as those binding the N-terminal domain, can bind Spike without neutralizing infectivity (235). Although rare, neutralizing antibodies that poorly mediate Fc effector functions have also been described, such as antibodies to the MPER region of HIV-1 and SIV Env (229, 230).

Several factors may influence the formation of antigen-IgG-Fc receptor complexes and subsequent effector cell activation. First, hydrogen-deuterium exchange (HDX) mass spectrometry and fluorescence correlation spectroscopy experiments demonstrated that IgG Fc domains undergo an allosteric conformational change in response to Fab antigen binding to facilitate FcɣR recognition (236). FcɣR binding and signaling lead to activation of Syk kinase which causes actin cytoskeletal rearrangements, allowing for subsequent Fc receptor clustering on macrophage membranes (237). Additionally, it is thought that the accessibility of the antibody Fc domain to Fc receptors is a critical geometric determinant to form these complexes (reviewed by (238)). In support of this hypothesis, it was shown that the antibody binding angle correlates with ADCC activity for several antibodies targeting the HIV-1 co-receptor binding site (239). Increasing Fc domain accessibility through lengthening the antibody hinge region can also increase phagocytosis of antigen coated beads (240). In addition to the binding orientation, Spike/Env tilting on the membrane may influence the Fc domain orientation and accessibility (103, 166). Antibody-mediated cross-linking of antigens can increase local Fc domain concentrations and tether viruses to cell surfaces, which may increase recognition by immune cells (157, 241). However, some monovalent antibodies (containing only one Fab domain) can outperform divalent IgGs at mediating Fc effector functions, suggesting that cross-linking does not universally enhance these processes (242). Monovalent antibodies may allow for more Fc domains to accumulate on the cell surface per multimeric antigen, thereby increasing FcR recognition (242). Altogether, the molecular basis for the formation of antigen-antibody-Fc receptor complexes in membranes needs further investigation.

### Fc effector functions against HIV-1

In the case of HIV-1, the importance of Fc effector functions for antiviral immunity is context dependent. It is important to distinguish the antibody antiviral activities between *preventing infection* (protection against viral acquisition) and *clearance* of virus-infected cells after infection. The contribution of Fc effector functions in *preventing* HIV-1 infection is not completely understood; several studies suggest that they are either a major contributor to protection or that their contribution is limited. First, in the human RV144 vaccine trial, a non-significant trend was found showing subjects with higher Fc effector functions had a lower risk of HIV-1 acquisition, which hinted that Fc effector functions may play a role in protection (243, 244). Additionally, an antibody with modest neutralizing potency and breadth, b12, exhibits significantly reduced protection compared to the LALA-variant (with reduced FcγR binding) in a rhesus macaque simian-human immunodeficiency virus (SHIV) infection model (245, 246). Further, changing the antibody IgG subclass enhances or reduces protection against vaginal HIV-1 acquisition in correspondence with the subclass affinity for Fc receptor in bone-marrow liver thymus (BLT) humanized mouse models (247). However, other studies have demonstrated little-to-no contribution of Fc effector functions in preventing viral acquisition. First, a LALA variant of potently neutralizing antibody, PGT121, afforded protection similar to the wild-type IgG in a rhesus macaque SHIV infection model, suggesting a limited contribution from Fc-effector functions (248). Additionally, afucosylated (with higher Fc-receptor affinity) b12 antibody did not significantly improve protection against SHIV challenge in macaques (249). Further, a very weakly neutralizing antibody with potent ADCC activity against SIV, PGT145, affords no protection in an SIV macaque infection model (147). In this study, partial protection was achieved using a SIV challenge stock containing a K180S mutation in Env that renders the virus sensitive to neutralization by PGT145, underscoring neutralization as a critical determinant of protection (147). Finally, several rhesus macaque studies have consistently shown limited protection afforded by non-neutralizing antibodies against SHIV challenge (250–252). In summary, while neutralization is a critical determinant of protection against HIV-1 viral acquisition, antibody Fc effector functions may in some cases augment the ability of neutralizing antibodies to mediate protection.

Although the role of Fc effector functions in protection against HIV-1/SIV acquisition continues to be debated, it is more widely accepted that these functions are essential for clearing virus-infected cells, including the long-lived reservoir of latently infected cells. Neutralizing activity mediated by antibodies or various compounds alone will not cure infection because HIV-1 integrates into host DNA and persists in latently infected cells (reviewed by (253)). Soon after cessation of anti-retroviral therapy (ART), the virus consistently rebounds within weeks (254). Thus, clearance of the viral reservoir through antibody Fc effector functions continues to be a major effort in the search for a widely implementable HIV-1 cure strategy (253). HIV-1 has evolved several mechanisms of resistance to Fc effector functions, such as limiting exposure of functional Env on virus-infected cells (255, 256), Vpu downmodulation of the host restriction factor tetherin to prevent accumulation of virions on the cell surface (257), and conformational masking of conserved epitopes by Nef/Vpu mediated CD4 downregulation. In the absence of Nef/Vpu, CD4 can bind the Env trimer on cell surfaces *in cis*, inducing an open conformation of the trimer and exposing vulnerable epitopes (258–261).

Many antibodies induced through HIV-1 infection are non-neutralizing antibodies which bind to the co-receptor binding site, as well as the conserved inner domains of gp120, likely elicited from shed gp120 subunits (261–266). These antibodies in HIV-1+ sera bind to epitopes normally conformationally occluded on functional, unliganded Env trimers, and therefore are generally ineffective at mediating Fc effector functions against productively infected cells (260, 261, 265). Small molecules which “open” the trimer and allow for convalescent sera to mediate Fc effector functions are being investigated (45, 166, 265, 267, 268). These small molecules bind gp120 in the Phe 43 cavity near the CD4-binding site and are therefore called “CD4 mimetic” (CD4mc) compounds (166, 267, 269, 270). It was shown that CD4 mimetic in combination with non-neutralizing antibodies or HIV+ sera significantly delay viral rebound and reduce viral reservoirs in humanized mice (271). The contribution of Fc effector functions in reducing viral reservoirs in this study was demonstrated using NK cell depletion or the use of LALA antibody with abrograted Fc receptor binding (271). Additionally, macaques immunized with HIV-1 gp120 antigen were significantly protected against CD4mc-sensitized SHIV challenge compared to macaques challenged with non-sensitized SHIVs (272). This underscores the protective synergy between CD4mc compounds and antibodies induced through vaccination (272). In addition to facilitating ADCC responses, CD4 mimetic compounds may reduce CD4+ T cell loss during HIV-1 infection. Uninfected bystander cells coated with shed gp120 antigen can be targeted for killing by Fc effector functions such as ADCC (234). CD4mc compounds block the CD4-binding site on shed gp120 and thereby prevent the coating of uninfected CD4+ bystander cells (265). Thus, CD4 mimetics in combination with antibodies, convalescent sera, or vaccination hold promise as an HIV-1 treatment or cure strategy though mediating clearance of persistent virus-infected cells and potentially reducing CD4^+^ T cell loss.

### Fc effector functions against SARS-CoV-2

Fc effector functions generally contribute to protection and/or viral clearance for SARS-CoV-2 infection. Although mutations in Spike have decreased the virus neutralization potency of monoclonal antibodies, vaccine-elicited sera, or convalescent sera, Fc effector functions of antibodies may be protective, more cross-reactive, and longer lasting (273–278). In fact, most of the antibodies induced through vaccination are Spike-binding, nonneutralizing antibodies (203, 279). The relatively high prevalence of nonneutralizing antibodies may be explained by the larger size of the SARS-CoV-2 Spike compared to HIV-1 Env. Spike presents more nonneutralizing epitopes which antibodies can bind without obstructing receptor interactions through steric occlusion. These Spike-binding antibodies are often capable of mediating Fc effector functions and arise early after vaccination. One dose of mRNA-1273 Spike vaccine demonstrated approximately >90% efficacy against SARS-CoV-2 infection after 14 days (280), whereas one dose of the BNT162b2 vaccine demonstrated >50% efficacy as early as 12 days after the first dose (281). Participant sera three weeks after one dose of the BNT162b2 vaccine exhibited relatively weak neutralizing activity but strong ADCC activity (274), suggesting that antibody Fc dependent functions may play a major role in protection during the development of early immune responses.

Murine models and Syrian hamster models of SARS-CoV-2 infection have been used to mechanistically probe the role of Fc effector functions in protection against SARS-CoV-2. Ullah et al. demonstrated that weakly neutralizing human convalescent sera with strong Fc effector function activity mediates protection against SARS-CoV-2 infection in prophylaxis and therapy using K18 ACE2 transgenic mice (278). The efficacies of these convalescent sera were highly dependent on the presence of effector cells during therapy (278). Additionally, mutations that abrogate Fc receptor binding or depletion of immune effector cells reduce protection mediated by neutralizing monoclonal antibodies during therapy and in some cases prophylaxis (282, 283). A nonneutralizing monoclonal antibody to the Spike NTD, DH1052, can also protect mice against lethal SARS-CoV-2 challenge, suggesting that Fc effector functions alone are in some cases sufficient for protection (235). Further, immunization of Fc receptor KO mice showed that Fc effector functions contribute to protection during active immunization (284). Altogether, mechanistic *in vivo* studies point towards a beneficial role for Fc effector functions in protection against SARS-CoV-2, and these functions become increasingly important during therapy for resolving infection. Given the documented importance of Fc-mediated immunity against SARS-CoV-2 infection, more research should be conducted on how to elicit and exploit these functions to provide more durable, cross-reactive immunity and augment neutralizing immunity.

## Discussion

Despite employing different envelope glycoproteins, HIV-1 Env and SARS-CoV-2 Spike share fundamental hallmarks of class I viral fusion proteins. Both rely on metastable prefusion states that refold through extended intermediates into compact postfusion conformations, with membrane fusion facilitated by the collapse of the prehairpin intermediate. These shared properties not only reveal common mechanistic vulnerabilities but also highlight why conserved regions of subunit 2 are attractive targets for therapeutic antibodies and vaccine design. Despite this conservation, these fusion subunits have evolved sophisticated immune evasion mechanisms. HIV-1’s extraordinary mutation rate, structural plasticity, and dense glycan shield continue to hinder the induction of durable broadly neutralizing antibodies. For SARS-CoV-2, vaccines have been highly effective in the induction of neutralizing antibodies and reducing severe disease, yet the emergence of immune-evasive variants demonstrating remarkable plasticity in the S1 subunit highlights the fragility of current immunization strategies.

Emerging advances in structural biology, cryo-EM, cryo-ET and molecular dynamics are beginning to resolve transient and elusive intermediates of the fusion process, providing unprecedented opportunities for rational design of immunogens that expose cryptic or transient epitopes. Fc effector functions add an additional layer of antiviral immunity, offering avenues for therapeutic antibody design and immunization strategies that extend beyond neutralization alone. Going forward, major priorities include: (1) stabilizing or mimicking fusion intermediates to better expose conserved epitopes; (2) designing mosaic or nanoparticle immunogens to broaden responses against divergent viral strains; and (3) harnessing Fc-mediated functions to clear virus-infected cells and latent reservoirs. By uniting structural and mechanistic insights with immunological strategies, it may be possible to develop durable vaccines and therapies against current and future pandemic threats.
